# Chronic PET‐Microplastic Exposure: Disruption of Gut–Liver Homeostasis and Risk of Hepatic Steatosis

**DOI:** 10.1002/advs.202512030

**Published:** 2025-10-22

**Authors:** Surye Park, Min‐Ji Kim, Jae‐Ho Shin, Eunsoo Kim, Myeongjoo Son, Seungjun Lee

**Affiliations:** ^1^ Department of Applied Biosciences Kyungpook National University Daegu 41566 Republic of Korea; ^2^ NGS Core Facility Kyungpook National University Daegu 41566 Republic of Korea; ^3^ College of Pharmacy and Research Institute for Drug Development Pusan National University Busan 46241 Republic of Korea; ^4^ Department of Anatomy & Cell Biology School of Medicine Kangwon National University Chuncheon 24341 Republic of Korea; ^5^ Brain Health Research Laboratory Institute of Medical Science Kangwon National University College of Medicine Chuncheon 24341 Republic of Korea

**Keywords:** dysbiosis, gut microbiota, gut–liver axis, non‐alcoholic fatty liver disease, polyethylene terephthalate

## Abstract

Microplastics (MPs) are pervasive pollutants found in environments and food, with humans experiencing continuous exposure. Polyethylene terephthalate (PET) is a major plastic contaminant detected in food and beverages. However, the chronic effects of environmentally relevant, physically abraded PET‐MPs on health, on the liver, remain elusive. This study investigates the hepatotoxicity of the PET‐MPs following long‐term exposure, with a focus on the gut–liver axis interactions and transcriptomic responses. Male mice are exposed to PET‐MPs (5 mg week^−1^) from 5 to 34 weeks of age. Environmentally mimetic PET particles are prepared by grinding plastic bottles. Prolonged exposure to MPs for 29 weeks led to increased adiposity and obesity. The mice exposed to PET‐MPs developed hepatomegaly, steatosis, and early‐stage fibrosis. Transcriptome data revealed the downregulation of genes involved in mitochondrial energy metabolism and the upregulation of lipid droplet and fibrotic pathway genes. Gut microbiota is also significantly changed, with a decrease in *Bacteroides* and *Lachnospiraceae*, which maintain gut homeostasis. Disruption of bile acid metabolism and fecal color change further indicated impairment of the gut–liver axis. These findings provide new toxicological insights and emphasize the requirement to reassess the public health risks posed by long‐term MPs ingestion.

## Graphical abstract

Chronic exposure to environmentally relevant PET microplastics disrupts gut–liver homeostasis, leading to hepatic steatosis, early fibrosis, and altered gut microbiota. These effects signal metabolic imbalance and gut–liver axis impairment, emphasizing chronic microplastic ingestion as an emerging environmental health risk linked to non‐communicable diseases with serious population‐level public health implications.

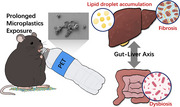

## 1 Introduction

The widespread use of plastics has resulted in the contamination of the environment and food with micro‐and nanoplastics. Humans are continuously and inevitably exposed to microplastics (MPs) through environmental and dietary pathways throughout their lifespan. Especially, polyethylene terephthalate (PET) is one of the most commonly used plastics and is widely used in bottled beverages, textiles, and food packaging.^[^ ^1^ ^]^ PET‐MP presence has been observed in various biological matrices, including human blood, feces, and placental tissues, raising concerns regarding their potential long‐term health effects.^[^ ^2^ ^]^ However, the toxicological effects of chronic exposure to PET/MP, especially on hepatic function, remain largely unknown.

Non‐alcoholic fatty liver disease (NAFLD) has emerged as a major global health issue, with its prevalence steadily rising, posing serious implications for healthcare systems worldwide. This condition encompasses a range of liver abnormalities, mainly involving fat accumulation in the hepatocytes of individuals with minimal or no alcohol intake.^[^ ^3^ ^]^ NAFLD's clinical spectrum ranges from simple steatosis, characterized by fat deposition without inflammation or tissue damage, to its more severe form, nonalcoholic steatohepatitis (NASH). NASH is a progressive subtype that involves hepatic inflammation and cellular injury that may lead to fibrosis, cirrhosis, and hepatocellular carcinoma. The majority of patients with NAFLD do not progress to NASH.^[^ ^4^ ^]^

Understanding the factors contributing to NAFLD is critical for developing effective prevention and management strategies. Metabolic, genetic, and environmental factors influence NAFLD, which is a multifactorial condition. Obesity has especially been identified as a predominant driver of hepatic fat accumulation via elevated adipose tissue and impaired metabolic regulation. Furthermore, obesity is strongly linked to NAFLD onset.^[^ ^5^ ^]^ Another primary factor is insulin resistance, which disrupts lipid homeostasis in the liver and exacerbates fat deposition, often along with type 2 diabetes mellitus.^[^ ^6^ ^]^ Dyslipidemia, characterized by increased triglyceride and low‐density lipoprotein cholesterol levels, further exacerbates NAFLD risk.^[^ ^7^ ^]^ Closely associated with these metabolic derangements is metabolic syndrome, a cluster of conditions that includes central obesity, increased blood glucose, and hypertension, which substantially increases the likelihood of NAFLD development.^[^ ^8^ ^]^ In addition to metabolic dysfunction, genetic predisposition plays a crucial role in NAFLD pathogenesis. Genetic variants, such as those in the patatin‐like phospholipase domain‐containing protein 3 gene, are strongly associated with disease progression. These genetic changes might elevate susceptibility by influencing hepatic fat metabolism and inflammation.^[^ ^9^ ^]^

Recently, the role of environmental factors in NAFLD onset and progression has gained traction. A pressing concern regarding MPs is their ubiquity in the environment. Although these effects may differ depending on the MP type, they have been increasingly implicated in adverse health effects, including liver toxicity. Previous studies using polystyrene (PS)‐MPs have demonstrated that PS‐MP exposure can induce oxidative stress by depleting antioxidant enzymes such as glutathione, catalase, and superoxide dismutase, while increasing levels of reactive oxygen species (ROS) and malondialdehyde. This oxidative imbalance, along with mitochondrial dysfunction, has been linked to hepatic cell damage. Moreover, PS‐MPs have been shown to elevate pro‐inflammatory cytokines (e.g., Tumor Necrosis Factor‐α, TNF‐α; Interleukin‐6, IL‐6; Interleukin‐1β, IL‐1β) and activate inflammatory signaling pathways such as NF‐κB, thereby aggravating liver inflammation and potentially accelerating NAFLD progression.^[^ ^10^, ^11^, ^12^ ^]^ Although MPs encompass a wide range of materials, including PS, polyethylene, polypropylene, and PET, most toxicological studies have focused on PS because of its availability and ease of use. Recent studies have shown notable differences in the toxic effects of different MP polymers.

Here, we aimed to address the critical gaps in the existing research by assessing the long‐term effects of PET‐MPs prepared using a novel method that mimics the irregular shapes and diverse physical properties of environmental MPs. Our approach reflects the heterogeneity of real‐world MPs and offers a more realistic toxicological assessment, unlike conventional studies, which use uniform spherical particles. Prolonged ingestion is required in animal models to overcome the limitations of short‐term toxicological studies. The long‐term exposure model facilitated the identification of chronic liver toxicity and systemic alterations, confirming that prolonged PET‐MP ingestion led to substantial hepatotoxic effects. This study provides a more accurate and representative assessment of microplastic‐related risk by integrating environmentally realistic particle preparation with a prolonged exposure framework. Our results highlight the criticality of adopting refined and realistic methodologies in future MP toxicology studies to better inform public health risk evaluations and regulatory decisions.

## 2 Results

### 2.1 Long‐Term MP Exposure Results in Increased Body Weight and Fat Mass in Mice

**Figure 1:**
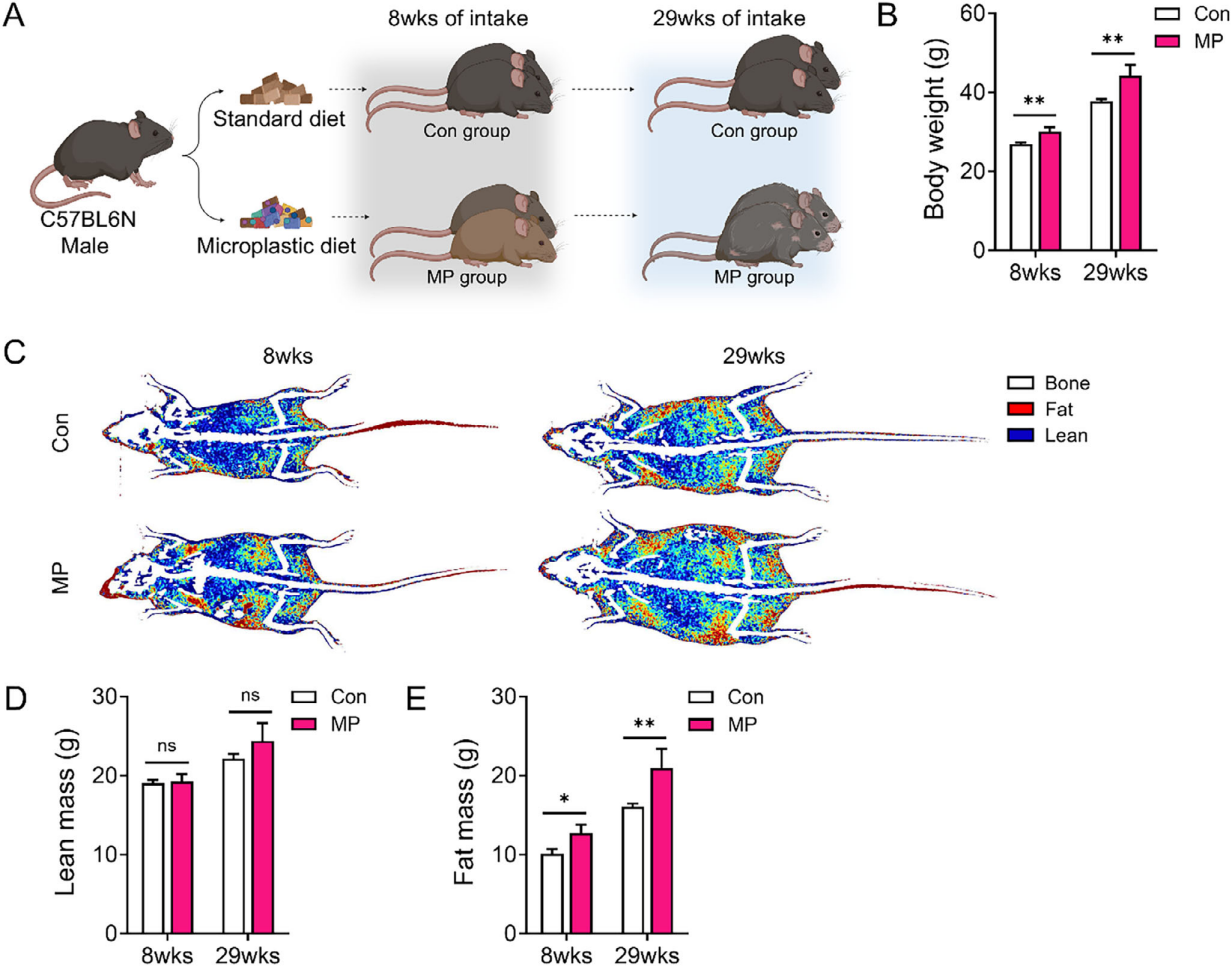
Phenotypic alterations in mice following MP ingestion at 8 and 29 weeks. A) The schematic illustrates mice fed either a control or MP diet for 8 or 29 weeks. Figure (A) was created using BioRender (https://biorender.com). B) Body weight (n = 33) was measured immediately after sacrifice. C–E) Body composition was measured in mice following MP ingestion for 8 and 29 weeks. Representative dual‐energy X‐ray absorptiometry (DXA) images from each group, with fat tissue shown in red and lean tissue in blue. Graphs show quantitative assessments from the DXA images: (D) lean mass, (E) fat mass of body weight. All data are presented as mean ± SEM. Statistical significance was evaluated using unpaired two‐tailed *t*‐tests between groups at each time point. Asterisks indicate statistically significant differences: ^*^ *p* < 0.05, ^**^ *p* < 0.01, ^***^ *p* < 0.001. Each experiment was independently repeated at least three times.

C57BL/6N male mice were randomly assigned to either the control (Con) group or the treatment group (MP group) via oral administration to investigate PET‐MPs’ effects. The MP group received 5 mg PET weekly for 29 weeks, with observations conducted at 8 and 29 weeks (**Figure** **1**A). High‐resolution scanning electron microscopy images confirmed the irregular morphology and surface roughness of the mechanically abraded PET microplastics used in this study (Figure S3, Supporting Information). The particles exhibited heterogeneous shapes with sharp edges and a broad size distribution. These morphological features may align with the intended environmentally relevant particle preparation. In addition, the distribution of PET‐MP sizes was confirmed using laser diffraction (Table S1, Supporting Information). The majority of PET‐MPs were within the 10–200 µm range, with ≈60% of the particles between 10–100 µm and 26% between 100–200 µm. Body weight and composition were analyzed at these time points. By week 29, mice in the MP group had substantially higher body weights than those in the Con group (Figure 1B). During the 8‐week study period, no considerable differences were observed in body weight between the groups. Radiographic DXA images showed differences in body fat distribution. Mice in the MP group showed larger red areas, indicative of higher fat density, compared to the blue and yellow areas, representing lower fat densities in mice in the Con group (Figure 1C). Quantitative DXA analysis confirmed these observations, revealing no change in lean mass or an increase in fat mass in the MP group (Figure 1D,E). These results highlight the chronic effect of MP ingestion on body composition, especially its role in promoting fat accumulation and changing fat distribution.^[^ ^13^ ^]^ Thus, long‐term MP ingestion substantially changes body composition by increasing body weight and fat mass, underscoring the metabolic risks linked to prolonged MP ingestion.

### 2.2 MP Ingestion Alters Gut Microbiota Composition and Impairs Intestinal Mucus Barrier Integrity

This study aimed to explore how orally administered MP influences gut microbiota composition and potentially affects liver function by assessing microbial shifts and their systemic effects. Principal coordinates analysis (PCoA)of the gut microbiota composition showed distinct clustering between the MP and Con groups at 8 and 29 weeks. At 8 weeks, the MP group demonstrated significant separation (Adonis R^2^ = 0.176, p = 0.006), indicating early changes in microbial composition. By week 29, the separation persisted, although the effect size was slightly decreased (Adonis R^2^ = 0.114, p = 0.028).

**Figure 2:**
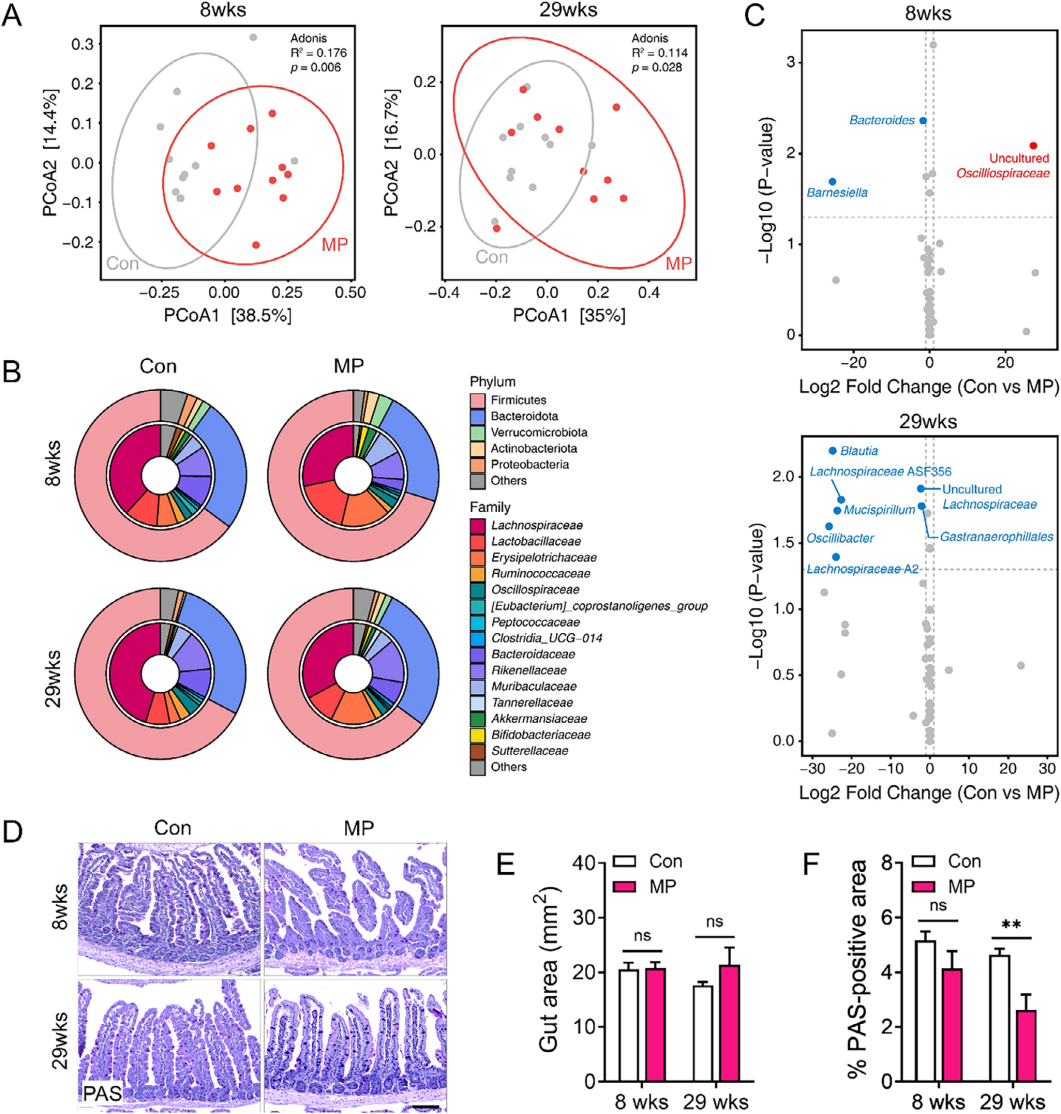
Microbial alterations and reduced intestinal mucus production in mice following MP ingestion at 8 and 29 weeks. A) Principal coordinates analysis (PCoA) of gut microbiota composition at 8 and 29 weeks based on Bray‐Curtis dissimilarity of genus‐level relative abundance. B) Donut plots represent the mean relative abundances at 8 and 29 weeks, with the outer and inner rings showing taxonomic composition at the phylum and family levels, respectively. C) Volcano plots show differentially abundant genera between the Con and MP groups at each time point. D) Representative images of gut tissue sections stained with periodic acid‐Schiff (PAS) reagent to visualize mucus production. Scale bar: 100 µm. E) Quantification of total gut sectioned area and mucus production, expressed as PAS‐positive area, and F) normalized with total in the gut was measured PAS‐positive area. Statistical significance was evaluated using unpaired two‐tailed *t*‐tests between groups at each time point. Asterisks indicate statistically significant differences: ^*^ *p* < 0.05, ^**^ *p* < 0.01, ^***^ *p* < 0.001. Each experiment was independently repeated at least three times.

Further analysis of microbial phylum distribution showed considerable differences in the relative abundances of the dominant phyla between the Con and MP groups. At 8 and 29 weeks, mice in the MP group showed an increased Firmicutes proportion (pink in the donut plot) and a decreased Bacteroidota proportion (blue in the donut plot) compared to the Con group (**Figure** **2**B). This shift suggests a potential link between MP ingestion and gut microbiota dysbiosis, which may contribute to metabolic disturbances such as altered lipid metabolism, increased energy extraction, and systemic inflammation.^[^ ^14^, ^15^ ^]^

A comparison of the relative abundances at the genus level showed substantial alterations in specific microbial genera. Notably, the relative abundance of *Bacteroides*, a genus involved in polysaccharide breakdown and gut homeostasis maintenance, was considerably lower in mice in the MP group at 8 and 29 weeks than in the Con group (Figure 2C and Figure S2, Supporting Information).^[^ ^16^ ^]^ Similarly, the abundance of several *Lachnospiraceae* genera, which produce short‐chain fatty acids (SCFAs) such as butyrate to support intestinal health, was reduced in the MP group (Figure 2C and Figure S2, Supporting Information).

To assess whether long‐term MPs ingestion affects intestinal mucus secretion and epithelial barrier integrity, we performed PAS staining on cross‐sections of the small intestine. Long‐term ingestion of MPs led to a noticeable reduction in intestinal mucus production, as evidenced by diminished PAS staining in the small intestine (Figure 2D–F). Compared to controls, MP ingested mice exhibited substantially decreased mucin accumulation in goblet cells. This reduction was more pronounced at 29 weeks, suggesting progressive disruption of mucosal integrity over time. These results suggest compromised mucus barrier integrity, which may reflect broader disturbances in gut homeostasis associated with chronic MP exposure.

Overall, these results illustrate the progressive and time‐dependent effects of MP exposure on the gut microbiota composition, with potential implications for systemic health. Furthermore, long‐term MP ingestion led to a noticeable reduction in intestinal mucus production, as evidenced by diminished PAS staining and decreased mucin accumulation in goblet cells, suggesting compromised mucus barrier integrity and progressive disruption of mucosal defenses. Additionally, a brighter color change was observed in the MP group compared to that in the Con group (Figure S1, Supporting Information). Although different factors, including diet and microbial metabolism, can influence fecal color, it is also known to reflect changes in the biliary and pancreatic systems. Given that bile pigments markedly contribute to normal fecal coloration, the observed brightening suggests that MP exposure influences bile secretion or flow. This raises the hypothesis that MP ingestion influences the hepatobiliary–pancreatic axis and may influence hepatobiliary function.

### 2.3 Long‐Term MP Ingestion Drives Severe Lipid Deposition and NAFLD Progression

**Figure 3:**
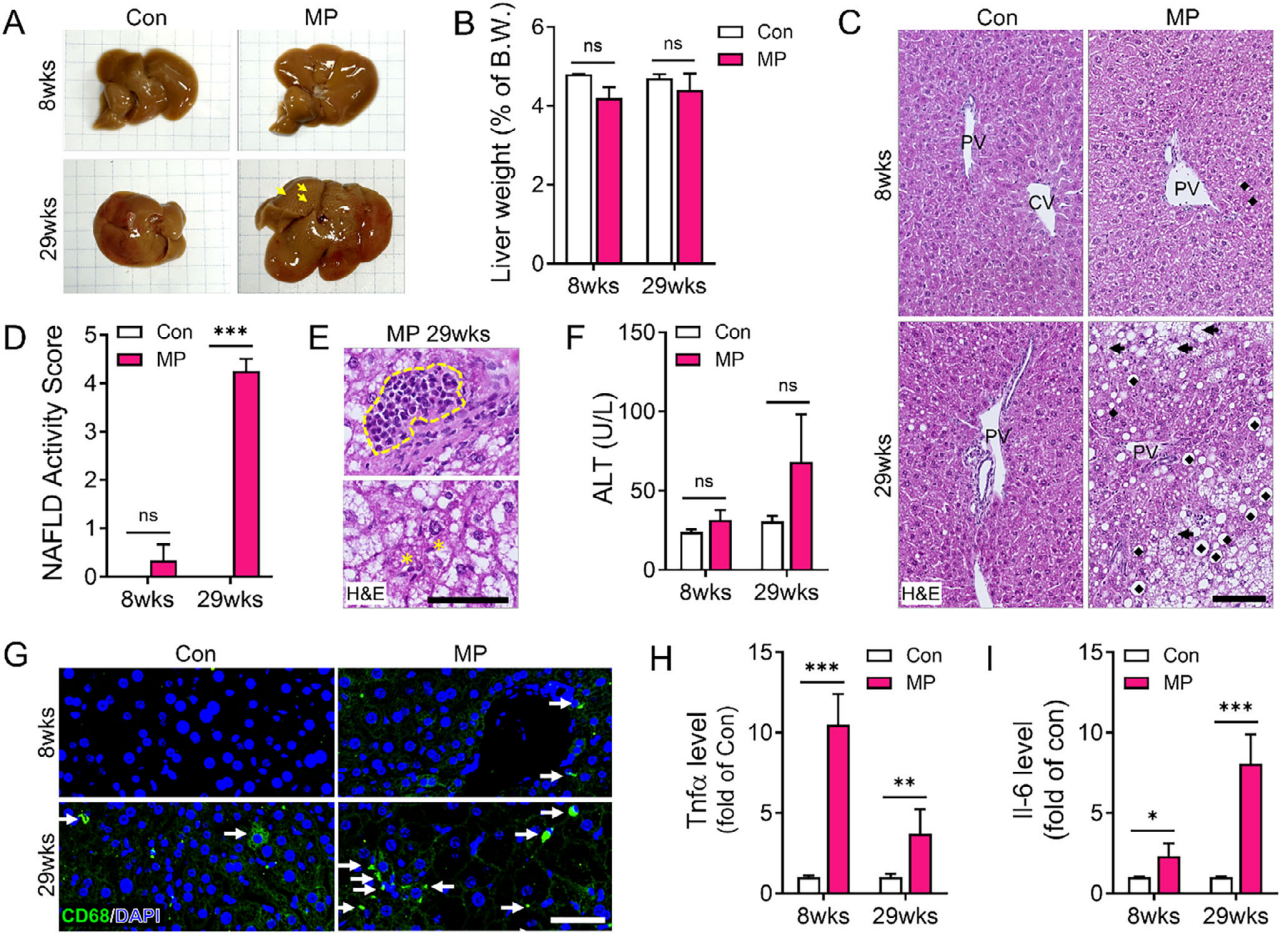
Histological, morphological, and inflammatory changes in the liver following MP ingestion. A) Representative liver images highlight morphological changes following MP ingestion. Yellow arrows indicate white areas observed on the liver surface. B) Liver weight (n = 21) was measured immediately after sacrifice. C) Hematoxylin and eosin (H&E) staining reveals hepatocyte swelling (arrow) and adipocyte accumulation (diamond). Scale bar: 100 µm. D) Nonalcoholic fatty liver disease (NAFLD) scoring demonstrates histological differences between Con and MP groups. Steatosis, lobular inflammation, and hepatocellular ballooning are graded based on the extent of lipid accumulation, number of inflammatory foci, and presence of ballooned hepatocytes, respectively. E) Higher‐magnification H&E images highlight eosinophil accumulation (upper) and Mallory–Denk bodies (lower, asterisk), which are cytoplasmic inclusions commonly observed in damaged hepatocytes. Scale bar: 60 µm. F) Blood alanine aminotransferase (ALT) (n = 13) levels increase in MP‐fed mice following MP ingestion. G) Representative immunofluorescence images of liver sections stained for CD68 (green) and DAPI (blue) from Con and MP groups at 8 and 29 weeks. White arrows indicate CD68‐positive macrophages. Scale bar: 50 µm. G) Representative immunofluorescence images of liver sections stained for CD68 (green) and DAPI (blue) from Con and MP groups at 8 and 29 weeks. White arrows indicate CD68‐positive macrophages. Scale bar: 50 µm. H, I) Quantification of hepatic mRNA expression levels of (H) Tnfα and (I) Il‐6, measured by qRT‐PCR and expressed as fold change relative to the corresponding control group at each time point. All data are presented as mean ± SEM. Statistical significance was evaluated using unpaired two‐tailed *t*‐tests between groups at each time point. Asterisks indicate statistically significant differences: ^*^ *p* < 0.05, ^**^ *p* < 0.01, ^***^ *p* < 0.001. Each experiment was independently repeated at least three times.

Gross examination of the liver showed clear morphological differences between the MP and Con groups. Notably, PET was not detected in liver tissues of the CON and MP groups by Py–GC/MS analysis targeting benzoic acid (Table S3, Supporting Information). At 29 weeks, the livers in the MP group showed irregular surfaces, discoloration, and visible white dots (highlighted by yellow arrows) indicative of fat accumulation. Contrastingly, the livers of the Con group mice were smooth and uniformly colored (**Figure** **3**A). Quantitative liver weight measurements revealed no statistically significant differences between the groups, suggesting that MP ingestion did not directly influence liver weight (Figure 3B). However, histological analysis using H&E staining showed pronounced changes in the livers of the MP group. They included ballooning hepatocytes, lipid accumulation (indicated by diamonds), inflammatory cell infiltration, and cytoplasmic clearing (indicated by arrows), which are features consistent with those of early‐stage NAFLD (Figure 3C). NAFLD was scored based on the histopathological results shown to assess the severity of liver damage. Mice in the MP group demonstrated substantially higher scores than those in the Con group, indicating increased steatosis, inflammation, and hepatocyte ballooning (Figure 3D). H&E staining showed the presence of Mallory–Denk bodies, indicating aggregated intermediate filaments and eosinophilic infiltration in the MP group. These features are hallmark indicators of severe liver injury and chronic inflammation (Figure 3E). Biochemical analysis of serum alanine aminotransferase (ALT) levels revealed a considerable elevation exclusively in the MP group at 29 weeks compared to the Con group. Elevated levels of ALT, a marker of hepatocellular damage, further supported the histological evidence of liver injury (Figure 3F). During NAFLD progression, inflammatory cytokines like TNF‐α and IL‐6 are known to increase. In our study, CD68 expression, a marker for macrophages, was assessed by immunohistochemistry, which revealed notable differences in the distribution and abundance of CD68‐positive macrophages between the MP and Con groups (Figure 3G). Hepatic mRNA levels of TNF‐α and IL‐6 were quantified by qRT‐PCR, confirming higher expression in the MP group compared to controls (Figure 3H, I). This suggests increased macrophage infiltration and activation (Figure 3G). Visual analysis showed a considerable increase in CD68‐positive macrophages in the MP group at 29 weeks compared to the Con group, indicating increased macrophage activity linked to long‐term MP ingestion (Figure S2A, Supporting Information). In line with increased CD68 staining, mRNA expression levels of inflammatory markers such as IL‐6 and TNFα were also substantially increased in the MP group at 29 weeks (Figure S2B, C, Supporting Information). These results suggest that MP‐induced macrophage activation contributes to a pro‐inflammatory microenvironment, potentially aggravating liver pathology (Figure 3).

Thus, these results demonstrated that long‐term MP ingestion induces substantial histopathological alterations in the liver, including NAFLD‐like features, increased inflammation, and hepatocyte injury. These alterations were corroborated by the corresponding increase in serum ALT levels, underscoring the systemic influence of prolonged MP ingestion.

### 2.4 Transcriptomic Analysis Shows Alterations in MP‐Induced Gene Expression and Metabolic Pathway

**Figure 4:**
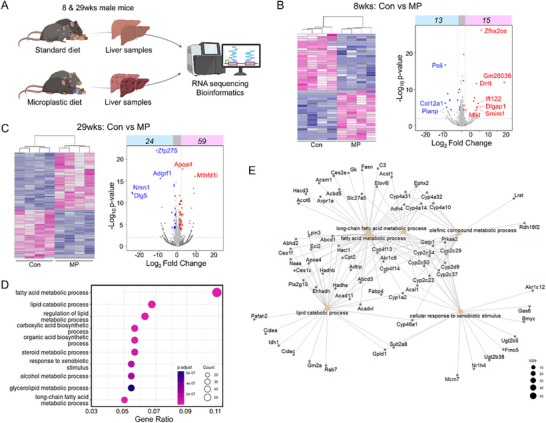
Liver transcriptomic changes following MP ingestion at 8 and 29 weeks. A) Schematic representation of mice fed either a control or MP diet for 8 or 29 weeks, followed by RNA sequencing of excised livers. Figure created with BioRender (). B, C) Heatmap and volcano plot of differentially expressed genes (DEGs), highlighting representative gene expression changes in the livers of MP ingestion mice. (B) shows DEGs from mice fed the MP diet for 8 weeks, while (C) shows DEGs from mice fed the MP diet for 29 weeks. The pink box (right) indicates upregulated genes, while the blue box (left) represents downregulated genes. Gene names are labeled. D) Gene Ontology (GO) enrichment analysis illustrates altered biological processes in MP ingestion mice at 29 weeks. E) Gene‐concept network visualizes the relationships between individual genes and biological concepts in MP ingestion mice at 29 weeks.

Building on the findings shown in Figure 3, which highlighted morphological and histological alterations in the liver tissue, RNA sequencing was conducted on livers from mice in the Con and MP groups at 8 and 29 weeks to investigate transcriptomic alterations induced by MP ingestion. Differential gene expression and functional enrichment analyses were conducted to identify the main pathways disrupted by long‐term MP ingestion (**Figure** **4**A). Volcano plots showed significant gene upregulation and downregulation in the livers of mice in the MP group compared with those in the Con group. At 8 weeks, 13 genes were markedly downregulated, and 15 were upregulated. The notable genes included *Poli*, *Col12a1*, and *Mki67*, which are involved in cellular metabolism and stress responses (Figure 4B). By 29 weeks, the number of differentially expressed genes substantially increased, with 24 downregulated and 59 upregulated genes. *Apoa4* and *Mthfd1l* stood out among the upregulated genes, as they are linked to lipid metabolism and one‐carbon metabolism, respectively (Figure 4C).

Gene Ontology (GO) enrichment analysis underscored MP‐induced changes in metabolic pathways. By 29 weeks, these effects became more pronounced, with substantial enrichment observed in pathways associated with lipid catabolism, fatty acid biosynthesis, and steroid metabolism. Progressive activation of lipid‐associated pathways highlighted the metabolic stress and lipid dysregulation caused by chronic MP exposure (Figure 4D). Representative gene‐concept network analysis showed the relationships among individual genes involved in processes such as fatty acid metabolism, long‐chain fatty acid metabolism, olefinic compound metabolism, lipid catabolism, and cellular responses to xenobiotic stimuli in MP‐ingesting mice at 29 weeks (Figure 4E). Thus, MP ingestion induced substantial transcriptomic alterations in the liver, especially affecting lipid metabolism and related pathways. These effects were more pronounced at 29 weeks, suggesting the cumulative effect of chronic exposure over time.

### 2.5 MP Ingestion Promotes Lipid Droplet Accumulation and Dysregulates LD‐Associated Gene Expression in the Liver

**Figure 5:**
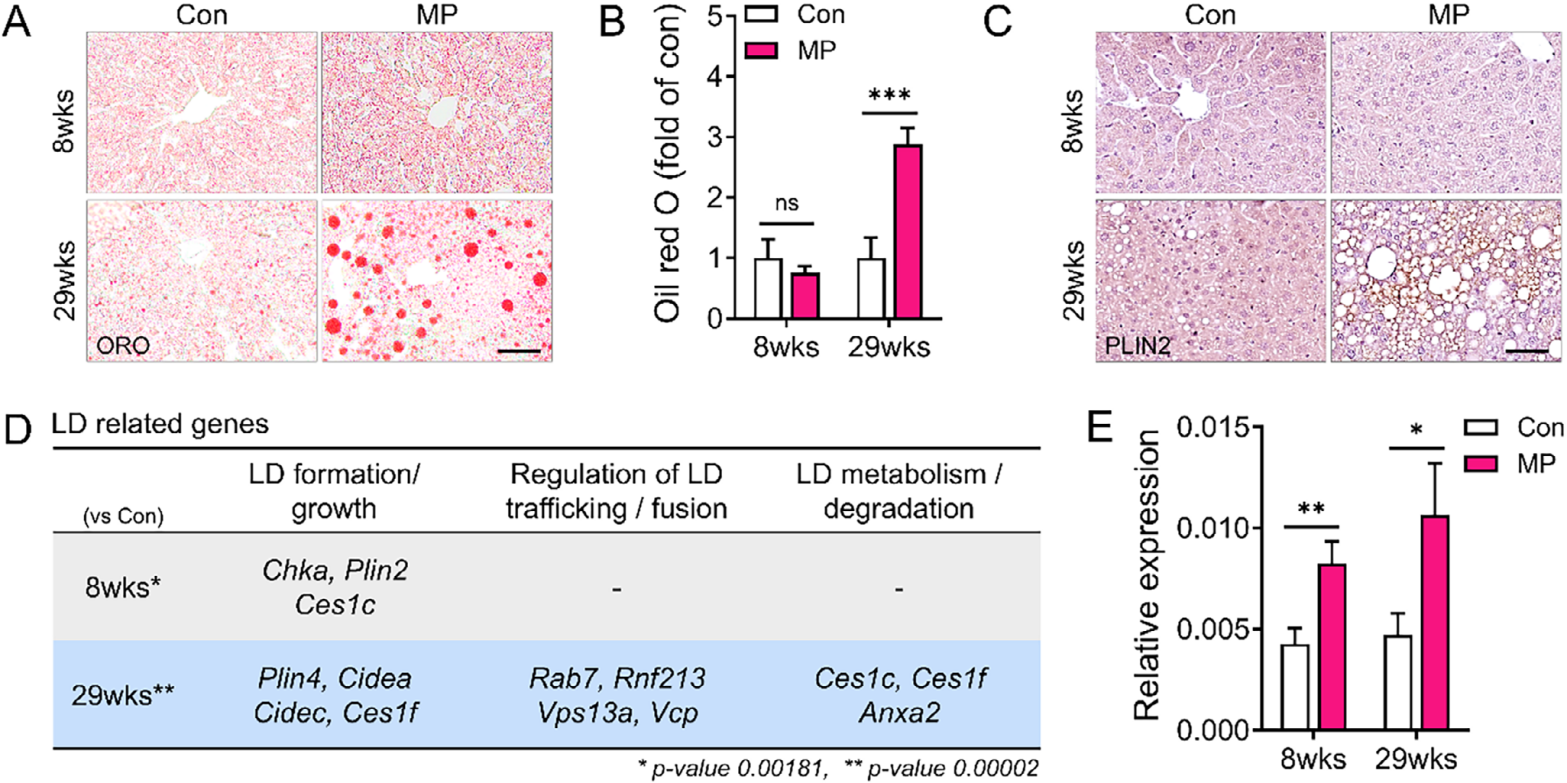
Increased lipid droplet accumulation following MP ingestion at 8 and 29 weeks. A) Microscopic images show Oil Red O (ORO)‐positive red droplets in liver tissue, and B) the corresponding graph shows the quantification of lipid deposition, measured using ImageJ software. C) Perilipin 2 (PLIN2), a protein associated with the surface of lipid droplets, is detected by immunohistochemistry. Scale bar = 50 µm D, E) RNA sequencing of liver samples reveals altered expression of lipid droplet (LD)‐related genes following MP ingestion at 8 and 29 weeks. Differentially expressed genes are labeled in (D). (E) Relative gene expression levels are shown as bar graphs. All data are presented as mean ± SEM. Statistical significance was evaluated using unpaired two‐tailed *t*‐tests between groups at each time point. Asterisks indicate statistically significant differences: ^*^ *p* < 0.05, ^**^ *p* < 0.01, ^***^ *p* < 0.001. Each experiment was independently repeated at least three times.

RNA sequencing of the liver showed broad genetic changes linked to lipid metabolism. We examined two key features of NAFLD to determine whether these transcriptional alterations were reflected in actual liver pathology: lipid accumulation and fibrosis. Oil Red O staining revealed that lipid droplet deposition (red dots) was the most prominent in the livers of mice after 29 weeks of MP ingestion (**Figure** **5**A, B). Additionally, we evaluated perilipin 2 (PLIN2) protein expression, which coats lipid droplets with phospholipids and facilitates neutral lipid storage. Immunohistochemical analysis showed a marked increase in PLIN2 protein expression in mice subjected to MP ingestion (Figure 5C).

Further analysis of the RNA sequencing data (Figure 4) showed MP‐induced transcriptional alterations in various genes associated with lipid droplet biology. In the 8‐week MP group, genes involved in lipid droplet formation and growth, such as *Chka*, *Plin2*, and *Ces1c*, were upregulated compared to those in the controls. In the 29‐week MP group, a broad range of lipid droplet‐related genes, including those involved in lipid droplet formation, growth, regulation, and metabolism, were upregulated (Figure 5D). Quantitative analysis confirmed that relative gene expression levels were elevated in the MP‐ingesting groups, with the most considerable alterations noted in the 29‐week ingestion group. Thus, transcriptomic and histological analyses consistently showed that prolonged MP ingestion resulted in elevated lipid accumulation and dysregulation of lipid droplet‐related genes in the liver (Figures 4 and 5).

### 2.6 Prolonged MP Ingestion Induces Liver Fibrosis and Upregulates Collagen and Extracellular Matrix (ECM)‐Related Gene Expression

**Figure 6:**
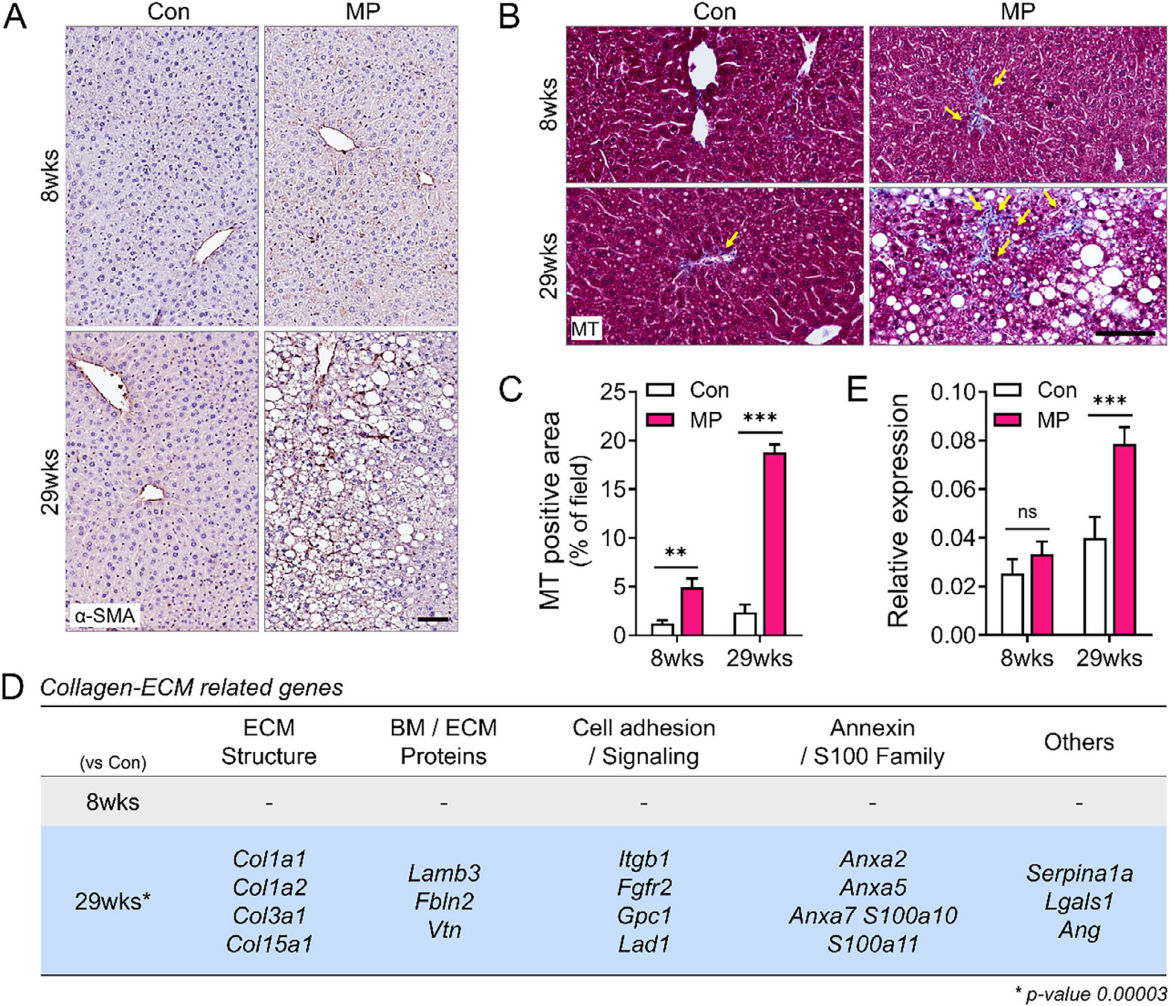
Increased fibrosis following MP ingestion at 8 and 29 weeks. A, B) Fibrosis markers are evaluated in liver tissue. (A) Microscopic images show α‐SMA expression (brown), and (B) Collagen fibers (blue) are visualized using Masson's trichrome (MT) staining. Fibrotic regions are indicated by yellow arrows, corresponding to blue‐stained areas in the MT image, respectively. Scale bars: 100 µm (B). C) Fibrotic area is quantified using ImageJ software based on representative MT‐stained images. D, E) RNA sequencing of liver samples reveals altered expression of collagen and extracellular matrix (ECM)‐related genes following MP ingestion at 8 and 29 weeks. Differentially expressed genes are labeled in (D). No significant changes are observed in the 8‐week group. (E) Relative gene expression levels are shown as bar graphs. All data are presented as Mean ± SEM. Statistical significance was evaluated using unpaired two‐tailed *t*‐tests between groups at each time point. Asterisks indicate statistically significant differences: ^*^ *p* < 0.05, ^**^ *p* < 0.01, ^***^ *p* < 0.001. Each experiment was independently repeated at least three times.

We performed immunohistochemical staining for α‐SMA to evaluate fibrosis progression in the liver. Positive α‐SMA signals (brown) were noted between lipid‐laden regions in the livers of mice after 29 weeks of MP ingestion, suggesting activation of the hepatic stellate cells (**Figure** **6**A). MT staining was performed to visualize collagen deposition (blue area) and further confirm fibrotic alterations. Blue‐stained collagen fibers were observed between the LD in the livers of MP‐ingesting mice (Figure 6B), indicating fibrotic progression. Quantitative analysis showed a substantial increase in the collagen‐positive area within the total field in the 29‐week treatment group (Figure 6C). Consistent with these histological findings (Figure 4), RNA sequencing data from the livers exhibited no notable alterations in collagen and ECM‐related gene expression at 8 weeks. Contrastingly, marked upregulation of collagen and ECM‐related genes was noted in the 29‐week MP ingestion group (Figure 6D, E). Fibrosis is closely linked to the upregulation of collagen‐ and ECM‐related genes, which contribute to tissue scarring and functional impairment.^[^ ^17^ ^]^ These results suggest that fibrotic alterations become apparent only following extended MP ingestion, even when lipid droplet accumulation begins as early as 8 weeks. This fibrotic response appears to result specifically from long‐term MP exposure rather than from time‐related processes. Thus, prolonged MP ingestion results in a distinct fibrotic response in the liver. This is characterized by increased α‐SMA expression, collagen deposition, and transcriptional upregulation of collagen and ECM‐related genes, which are not observed at earlier time points or in the Con group.

## 3 Discussion

MPs have rapidly emerged as a global environmental concern owing to their ubiquity in water, soil, air, and even the food chain.^[^ ^18^ ^]^ Although growing evidence of their potential hazards exists, previous studies have often treated MPs as a homogeneous group, overlooking the fact that polymer composition and particle shape can substantially influence toxicity profiles once ingested.

MP classification typically considers their origin, polymer composition, and morphology, which are factors that influence their fate, transport, and toxicological potential. Although in vitro studies have limitations in replicating real‐world human exposure, they consistently reveal that the specific polymer type of MP plays a crucial role in determining toxicological outcomes. A recent study^[^ ^19^ ^]^ underscored substantial toxicity differences among MP polymers, including PET, polyvinyl chloride (PVC), and PS in HepG2 liver cells. Especially, PET and PVC nanoparticles showed considerable cytotoxicity even at low concentrations, inducing oxidative damage that resulted in mitochondrial apoptosis. Contrastingly, PS nanoparticles did not induce similar severe effects, suggesting that the polymer density and chemical structure are the main toxicity determinants. These results raise growing concerns regarding PET, the main material in plastic water bottles that are commonly consumed worldwide. Although much of the existing literature focuses on polymers, such as PE and PS, PET remains understudied despite its widespread use.

In addition to the chemical composition, MP morphology, especially their shape, substantially influences their toxicological profile. A recent study comparing high‐density PE microbeads and irregularly ground low‐density PE fragments showed that rough, sharp edges substantially increased cytotoxicity, pro‐inflammatory cytokine release, and hemolysis, and these effects were less pronounced with smoother microbeads.^[^ ^20^ ^]^ These results emphasize that the irregular shapes that are generally found in real‐world MP, which arise from random degradation under physical and biological stressors, may pose more substantial health risks than initially anticipated.

In the present study, we aimed to simulate realistic exposure conditions by focusing on PET. We processed PET using a blender to produce irregularly shaped MPs, which were subsequently administered over an extended period. Our main objective was to investigate how PET particles influence the gut–liver axis, a complex, bidirectional communication system that links the gastrointestinal tract and liver via the portal vein, bile acids, and immune metabolic signaling pathways. This axis plays a central role in the maintenance of systemic homeostasis. Disruptions in gut microbial balance or barrier function can provoke hepatic inflammation and metabolic dysregulation. In turn, liver‐derived signals can affect intestinal physiology. Given this interdependence, understanding how PET‐derived MPs interact with the gut–liver axis is critical for elucidating their potential role in the pathogenesis of liver diseases.^[^ ^21^ ^]^

For example, ingested MPs mainly interact with the gastrointestinal tract and change the gut microbial community, resulting in dysbiosis. Mice exposed to these particles showed a substantial increase in Firmicutes and a decrease in Bacteroidetes, suggesting a shift toward an imbalanced microbiota (Figure 2). At the family level, *Bacteroidaceae* and *Lachnospiraceae* declined, whereas *Sutterellaceae* also consistently decreased. These alterations imply that long‐term MP ingestion can destabilize the gut microbial composition, potentially driving metabolic and immune changes over time. Particularly, when the Firmicutes/Bacteroidota balance shifts, especially if Firmicutes become predominant, an increased risk of obesity, metabolic syndrome, and systemic inflammation is suggested to occur.^[^ ^22^ ^]^ Specifically, at the genus level, Bacteroides and several genera belonging to the *Lachnospiraceae* family declined in MP exposed mice (Figure 2C). *Bacteroides* and *Lachnospiraceae* play a role in polysaccharide degradation and SCFA production and help to maintain intestinal barrier integrity.^[^ ^16^, ^23^ ^]^ These alterations imply that long‐term MP ingestion can destabilize the gut microbial composition, potentially driving metabolic and immune changes over time. For instance, this dysbiotic profile promotes increased intestinal permeability, facilitating the translocation of bacterial lipopolysaccharides and pro‐inflammatory mediators into portal circulation.^[^ ^24^ ^]^ The resulting endotoxemia can activate hepatic Kupffer cells and stellate cells, directly contributing to liver pathology through the disrupted gut‐liver axis.^[^ ^24^, ^25^ ^]^ Therefore, preservation of this ratio is crucial for optimal gut function and overall health. Our main objective was to assess how irregular PET MPs influence the gut–liver axis, a bidirectional communication network linking the gastrointestinal tract and liver via the portal vein, bile acids, and complex immune and metabolic signaling pathways. This axis is necessary for maintaining systemic health, as disruptions in the gut environment can influence liver function, and vice versa. Given that ingested MPs first interact with the gastrointestinal tract, they can change the composition of the gut microbiota and induce dysbiosis, an imbalance in microbial populations and functions (Figure 2B, C). Our results suggest that prolonged exposure to irregular PET MP may trigger dysbiosis, which can impair liver function and disrupt metabolic homeostasis. Understanding these interconnected processes is crucial for advancing comprehensive risk assessments and developing strategies to mitigate the potential adverse health effects of MP exposure. These disruptions, especially within the gut–liver axis, are increasingly recognized as contributing factors to the onset and progression of liver‐related diseases.

Recent research has increasingly underscored the potential link between MP exposure and NAFLD development. MPs have been indicated to impair hepatic fatty acid β‐oxidation and induce oxidative stress, contributing to NAFLD‐associated phenotypes.^[^ ^26^ ^]^ Moreover, prolonged exposure triggers cellular senescence, ferroptosis, and hepatotoxicity.^[^ ^27^ ^]^ In a zebrafish model, exposure to polystyrene MP combined with antibiotic residues increased lipid accumulation and liver inflammation.^[^ ^28^ ^]^ Although most of these investigations focused on polymers other than PET, the consistent observation of NAFLD outcomes underscores MP's hepatotoxic potential, regardless of the polymer type. MP exposure has also been implicated in amplifying oxidative stress via increased ROS, which activate pro‐fibrotic signaling pathways such as TGF‐β/Smad2/3, cGAS/STING, and NF‐κB. Co‐exposure to toxicants such as cadmium further exacerbates liver damage by promoting extracellular ATP release and hepatic stellate cell activation, thereby accelerating fibrosis. A retrospective analysis showed that MP polymers were detected in the livers of patients with cirrhosis, whereas no such findings were reported in individuals without liver disease.^[^ ^29^ ^]^

At the molecular level, MP exposure changes the pathways involved in PPAR signaling, oxidative stress, and complement and coagulation cascades. Studies using human pluripotent stem cell‐derived liver organoids have further revealed that MPs upregulate hepatic HNF4A and CYP2E1, key regulators of lipid metabolism, insulin signaling, and mitochondrial function.^[^ ^30^ ^]^ Our results similarly showed that prolonged MP ingestion results in more pronounced NAFLD‐like alterations in the liver tissues. RNA sequencing demonstrated substantial changes in the expression of genes linked to lipid metabolism (Figure 5), ECM structure, and cell adhesion (Figure 6). Notably, the mice subjected to extended exposure also showed mild cholestasis (Figure S1, Supporting Information) and alterations in fecal coloration, suggesting that long‐term MP consumption may exert systemic effects.

A key limitation of this study is the lack of detailed mechanistic exploration at the cellular level. While our in vivo model allowed us to assess the chronic systemic effects of PET‐MPs, it does not fully clarify how PET‐MPs initiate hepatocellular injury and metabolic disruption. Importantly, prior in vitro studies using HepG2 cells have shown that PET‐MPs can induce oxidative stress, impair mitochondrial DNA integrity, decrease mitochondrial membrane potential, and activate autophagy pathways.^[^ ^30^ ^]^ These processes are closely linked to hepatic fibrosis, as oxidative stress promotes stellate cell activation and extracellular matrix deposition^[^ ^31^ ^]^ and also to adipogenesis via altered lipid metabolism and mitochondrial signaling.^[^ ^32^ ^]^ In addition, our study is that it does not fully clarify the molecular mechanisms by which PET‐MPs induce hepatocellular injury and disturb gut–liver axis homeostasis. Histological and transcriptomic analyses confirmed NAFLD‐like pathology and inflammation, and our observation of gut microbiota dysbiosis with elevated TNF‐α and IL‐6 supports a mechanism involving increased intestinal permeability and translocation of pro‐inflammatory microbial products such as LPS. Prior work, largely on PS‐MPs, has implicated oxidative stress, mitochondrial dysfunction, and ferroptosis as key contributors to MP‐induced liver injury,^[^ ^31^, ^32^, ^33^ ^]^ and these processes may also operate in PET‐MP exposure. We will address this in future studies through targeted assays for oxidative stress markers (MDA, GSH/GSSG), circulating LPS, and ferroptosis‐related proteins (GPX4, ACSL4). In addition, our use of male C57BL/6N mice improved internal validity and comparability with existing hepatology/metabolism datasets but limits generalizability due to strain‐specific immune/metabolic features and the exclusion of females. To address this, future studies will include F1 hybrids and genetically diverse cohorts (e.g., Diversity Outbred, Collaborative Cross–derived) as well as balanced male–female designs with estrous‐aware sampling to assess sex‐ and genotype‐dependent effects under harmonized, contamination‐controlled conditions.

This investigation was designed as a hazard‐focused study rather than a pharmacokinetic/toxicokinetic analysis; polymer‐specific in‐tissue detection, biodistribution, and kinetic profiling were not performed. PET was not detected in the liver tissues of the MP group by Pyro‐GC/MS analysis. MPs may remain mobile in vivo, undergo progressive fragmentation, or degrade over time, making single‐point tissue measurements insufficient to capture their dynamic fate. We also noted qualitative fecal color changes following PET‐MP exposure, but without polymer‐specific quantification or degradation product analysis, excretion kinetics cannot be inferred. Quantitative post‐exposure kinetics (half‐life and clearance curves) of orally ingested PET‐MPs in mammals remain poorly defined in the current literature. Nevertheless, after one week of repeated oral dosing (≤10 µm), persistent gastric signals ≥24 h after each administration and ex vivo detection in the stomach and lungs have been reported, indicating incomplete short‐term clearance and potential organ residence (Kim et al., 2025). In view of these limitations, we interpret our findings as hepatic alterations associated with prolonged PET‐MP exposure, while acknowledging that definitive evidence of direct particle accumulation in the liver remains to be established. Importantly, our study provides one of the first long‐term in vivo demonstrations of PET‐MP‐induced hepatic pathology, and it highlights the need for future research to establish time‐resolved tissue burdens through direct tissue analysis combined with correlative spectroscopy.

Future work will use contamination‐controlled protocols to quantify fecal PET‐MPs via µFTIR/µRaman and pyrolysis–GC/MS, and to measure PET‐derived products (TPA, MHET, BHET) by targeted LC–MS/MS, integrating these results with mass‐balance calculations.

In‐tissue detection remains essential for linking particle presence to pathology. We therefore plan to combine µFTIR spatial mapping with pyrolysis–GC/MS quantification, standardized digestion and density separation, orthogonal spectral validation, procedural blanks, and spike–recovery controls. This comprehensive workflow will define PET‐MP accumulation, redistribution, and degradation patterns, thereby strengthening the mechanistic understanding of their role in NAFLD progression.

Biodegradable alternatives such as polylactic acid (PLA) and polyhydroxyalkanoates (PHA) are gaining traction because MPs continue to raise environmental and health concerns. Although these materials have the potential to reduce plastic pollution, their safety and environmental impact remain uncertain.^[^ ^34^ ^]^ PLA production is rapidly expanding, and although PLA is biodegradable, studies have suggested that PLA nanoparticles may cause intestinal inflammation, oxidative stress, and mitochondrial damage.^[^ ^35^, ^36^, ^37^, ^38^ ^]^ PHA, a bio‐based polymer capable of degradation in marine settings, is considered biocompatible; however, its breakdown can be extremely slow, potentially taking centuries under certain conditions.^[^ ^39^ ^]^ These results highlight the requirement for improved polymer designs, better waste management, and a deeper understanding of the long‐term environmental impacts of biodegradable plastics.^[^ ^40^ ^]^

Future research should focus on elucidating the toxicological mechanisms of various MP types, developing biocompatible polymers with faster degradation rates, and advancing detection technologies to trace MP across diverse environments to effectively mitigate MP pollution. Further studies are required to evaluate human exposure routes and long‐term health risks, especially in vulnerable populations.

## 4 Conclusion

MPs are persistent pollutants that have become an emerging threat to both the environment and human health. Their durability means that materials produced and discarded in past decades continue to circulate in ecosystems, with the potential to affect not only current but also future generations. This intergenerational dimension underscores the urgent need to understand the long‐term consequences of MP exposure.

In our research program, we first demonstrated that chronic ingestion of PET‐MPs impairs reproductive function, particularly testicular growth and maturation, with implications for fertility. This study focused on another critical organ, the liver, which plays a central role in metabolism, detoxification, and immune regulation. Our findings indicate that prolonged PET‐MP ingestion induces pathological changes consistent with NAFLD, accompanied by inflammation and disruption of the gut–liver axis. These results highlight the possibility that MPs act as hidden drivers of metabolic and chronic diseases, particularly when exposure is sustained over long timescales. The broader implications of this work extend beyond laboratory models. As MPs accumulate and fragment in the environment, they remain available for human exposure through food, water, and air. Their persistence ensures that today's contamination will not be confined to the present but will inevitably affect generations to come, who will inherit both the materials themselves and their biological consequences. Our findings emphasize the importance of hazard‐focused studies as a foundation for translational research. Future work should integrate toxicokinetic analyses, polymer‐specific detection methods, and genetically diverse, sex‐balanced animal models to enhance human relevance. By advancing this evidence base, we aim to inform not only scientific understanding but also regulatory and societal action. Ultimately, addressing MP pollution is not merely an ecological necessity but a public health imperative to protect both current and future generations.

## 5 Experimental Section

### Mouse Husbandry and Experimental Groups

A four‐week‐old male C57BL/6N mice (total number of mice: 33, mean initial body weight: 18–19 g) were used. Male C57BL/6N mice were selected to align with extensive historical control data and standardized husbandry conditions in our facility, enabling direct comparison with previous liver toxicology and metabolic studies. The use of males minimized variability from cyclical ovarian hormone fluctuations, thereby increasing sensitivity to detect the modest, chronic effects anticipated from environmentally relevant PET‐MP exposure. Seventeen mice were assigned to the control group and 16 mice to the PET‐MP ingestion group. At 8 weeks of feeding, 8 mice from each group were sacrificed, and at 29 weeks, the remaining animals were sacrificed, resulting in 8 mice in the 8‐week control group, 8 mice in the 8‐week MP group, 9 mice in the 29‐week control group, and 8 mice in the 29‐week MP group. Mice were obtained from Samtako Bio‐Korea Inc. (South Korea) and acclimated to the experimental environment for 7 days before the study. The mice were raised for 29 weeks and sacrificed at 33 weeks of age for further analysis, with all procedures performed in accordance with institutional animal ethics guidelines to minimize pain and distress. They were housed in a facility maintained at 25 ± 2 °C and 50 ± 5% relative humidity with a 12‐h light‐dark cycle (lights on from 07:00 to 19:00). The mice were provided chow and sterilized water ad libitum throughout the study. Furthermore, they were randomly assigned to groups at the start of the experiment, based on the balanced initial body weights. The experimental group (designated as “MP” in this study) ingested PET‐MP incorporated into their feed at a dose of 5 mg per week for 29 weeks. The PET‐MP dose (5 mg week^−1^) was determined by adjusting the reported human global average rate of Microplastic intake (GARMI, 5 g week^−1^) based on the human‐to‐mouse body weight ratio. Mice that did not ingest the PET‐MPs were designated as the control (Con) group.

The body weights (n = 33) of the mice were recorded weekly throughout the experimental period to monitor their health and evaluate the effects of PET‐MP exposure. Body composition and bone‐related parameters (n = 33) were measured using the InAlyzer system (Medikors, Korea), a dual‐energy X‐ray absorptiometry (DXA)‐based device.

All the animal experiments were conducted in accordance with the National Guidelines for the Care and Use of Laboratory Animals in Korea. The Institutional Animal Care and Use Committee of the Pukyong National University approved this study (permit no. PKNUIACUC‐2024‐45).

### PET‐MP Preparation and Characterization

New PET drinking‐water bottles (Samdasoo, Korea) were processed to minimize microbial and particulate contamination. Labels and caps were removed; bottles were triple‐rinsed with 18.2 MΩ·cm water, immersed in 70% ethanol for 10 min, and transferred to an ISO Class 5 laminar‐flow cabinet to air‐dry on pre‐combusted aluminum foil. Dried bottles were then UV‐C irradiated (254 nm) for 30 min per side immediately prior to grinding. High‐temperature/autoclave sterilization was not used to avoid PET deformation or hydrolysis. The stainless‐steel mill chamber and sieves were cleaned by detergent sonication, ultrapure‐water rinse, 70% ethanol wipe, and UV‐C exposure before use. All handling was conducted with powder‐free nitrile gloves and cotton lab coats; work surfaces were routinely wiped with 70% ethanol. PET‐MPs were prepared by grinding sterile water bottles, followed by sieving them through a stainless steel mesh with a 500‐µm pore size to obtain particles < 500 µm. A subset of these particles (< 500 µm) was collected, and 1 g of the sample was weighed for particle size analysis, which was conducted using a Malvern Panalytical particle size analyzer (Mastersizer 3000; Malvern, UK) in dry dispersion mode with an air pressure of 3 bar (gauge). Particle size analysis was conducted in triplicate to ensure precision. For imaging, focused ion beam‐scanning electron microscopy serial surface view imaging was performed using a Zeiss Crossbeam 550 dual‐beam microscope (Zeiss Microscopy GmbH, Oberkochen, Germany). The MP samples were elevated to a 5‐mm height, corresponding to the coincident point of the two beams, and tilted at 54°. The analyses of the scanning electron microscopy were independently repeated thrice to ensure reproducibility.

### Histological Morphometric Analysis

All the liver tissue samples were prepared for histological analysis using standard protocols. For Oil Red O staining, tissues were rapidly frozen to form blocks, which were cryosectioned at 10‐µm thickness. For other staining methods, including hematoxylin and eosin (H&E), Masson's trichrome (MT), Periodic acid‐Schiff (PAS), and immunohistochemistry, tissues were fixed in 10% neutral‐buffered formalin, embedded in paraffin blocks, and sectioned at 5‐µm thickness using a microtome. Before staining, paraffin was removed from the sections using xylene, and the tissues were rehydrated using graded ethanol solutions. The stained slides were examined under a light microscope (Olympus Optical, Tokyo, Japan) and scanned using a slide scanner for streamlined observation and histological quantification.

### Histological Morphometric Analysis—H&E and NAFLD Activity Scoring

Deparaffinized sections were rinsed with tap water and stained with hematoxylin (Merck, MO, USA) for 1 min to visualize the nuclei blue. The slides were stained with eosin Y (FUJIFILM Wako, Osaka, Japan) solution for 30 s to impart a red–pink hue to the cytoplasmic and extracellular components. After staining, the slides were dehydrated, cleared, and mounted with a xylene‐based DPX mountant (Fisher Scientific, NJ, USA) before being covered with coverslips.

To confirm liver damage severity, an experienced pathologist scored the NAFLD activity in a blinded manner according to the criteria established by the Non‐alcoholic Steatohepatitis Clinical Research Network.^[^ ^13^ ^]^ The NAFLD activity score yields a total score ranging from 0 to 8. Steatosis was graded based on the hepatocyte percentage containing lipid droplets: 0 (< 5%), 1 (5–33%), 2 (34–66%), and 3 (> 66%). Lobular inflammation was evaluated by counting the number of inflammatory foci per field as follows: 0 (none), 1 (< 2 foci), 2 (2–4 foci), and 3 (> 4 foci). Hepatocellular ballooning was scored as 0 (none), 1 (a few ballooned cells), or 2 (several cells or prominent ballooning).

### Histological Morphometric Analysis—MT for Fibrosis

Deparaffinized sections were re‐fixed in Bouin's solution for 24 h to enhance the staining quality and thoroughly rinsed with tap water. Sections were stained with Weigert's iron hematoxylin for 10 min to visualize the nuclei, followed by Biebrich scarlet acid fuchsin for 15 min to highlight the cytoplasmic elements. Differentiation was achieved using a phosphomolybdic–phosphotungstic acid solution for 10 min to remove excess dye. Sections were subsequently stained with aniline blue for 3 min to color the collagen fibers blue and washed with water. After staining, the slides were dehydrated, cleared, and mounted. Staining was performed according to the protocol provided with the MT Staining Kit (Bioquochem, Asturias, Spain). This technique allowed for the differentiation of liver tissue components, with cytoplasmic elements appearing red and collagen fibers appearing blue.

### Histological Morphometric Analysis—Oil Red O for Lipid Droplet Accumulation

Frozen liver tissues were cryosectioned at 10‐µm thickness, mounted on glass slides, and air‐dried. Sections were rinsed with distilled water to remove excess fixative, immersed in 70% ethanol, and stained with Oil Red O solution (Sigma‐Aldrich, MO, USA) for 15 min to visualize the lipid droplets. Excess stain was gently washed off with distilled water, and the sections were briefly counterstained with hematoxylin to highlight the nuclei. Finally, the slides were mounted using glycerol as an aqueous mounting medium and covered using coverslips. The prepared slides were examined under a light microscope (Olympus Optical) to detect the neutral lipids, which were visible as red‐stained lipid droplets in the liver tissue.

### Histological Morphometric Analysis—PAS for Intestinal Homeostasis and Mucus Production

To evaluate intestinal mucus production and barrier integrity, small intestine tissues were prepared in a Swiss roll configuration and sectioned at 5 µm thickness. Periodic acid‐Schiff (PAS) staining was performed to visualize mucin‐producing goblet cells. Whole‐slide images were acquired using an Axio Scan Z1 digital slide scanner (ZEISS, Oberkochen, Germany), and mucus production was quantified using QuPath software (v0.5.1). PAS‐positive areas were computationally identified and expressed as both absolute values and percentages of the total tissue area to assess relative mucus abundance across groups.

### Immunohistochemistry

Immunohistochemical analysis was conducted using an ABC Kit (Vector Laboratories). Deparaffinized sections were incubated with 0.3% hydrogen peroxide solution (Merck) for 10 min. After thorough rinsing with phosphate‐buffered saline (PBS), the sections were incubated with normal animal serum from an ABC kit at room temperature for 1 h to decrease nonspecific binding.

The sections were separately incubated with anti‐α‐smooth muscle actin (α‐SMA; cat. 14395‐1) and perilipin 2 (PLIN2; cat. 15294‐1) primary antibodies (Proteintech, IL, USA) at 4 °C overnight. A biotinylated goat anti‐mouse IgG secondary antibody was applied after three washes with PBS, followed by incubation with the avidin–biotin complex from the ABC kit. Visualization was achieved using 3,3′‐diaminobenzidine (DAB, Sigma‐Aldrich) as the chromogen for 5 min. Nuclei were counterstained with hematoxylin to provide a blue contrast. After staining, the slides were dehydrated, cleared, and mounted using DPX mounting solution (Fisher Scientific) before being covered with coverslips. The stained sections were examined under a light microscope (Olympus Optical), allowing for α‐SMA localization and PLIN2 expression within the liver tissue.

Following the DAB staining, which revealed the distribution of inflammatory cells, immunofluorescence staining was performed to further characterize specific cell populations in the liver tissue. After deparaffinization, rehydration, and antigen retrieval, slides were blocked with normal serum for 1 h at room temperature. They were then incubated overnight at 4 °C with an anti‐CD68 antibody (Abcam, MA, USA) to label macrophages. After thorough washing, a rabbit Alexa Fluor 488–conjugated secondary antibody (Abcam) was applied for 1 h at room temperature in the dark. Nuclei were counterstained with DAPI, and slides were mounted using VECTASHIELD mounting medium (Vector Laboratories). Imaging was performed with an Olympus FV3000 system, providing clear visualization of CD68‐positive cells within the liver tissue.

### Transcriptomic Analysis and Bioinformatics

Total RNA was extracted from mouse liver samples (n = 4 per group) using the RNeasy Mini Kit (Cat. no.74104; Qiagen, Germany) according to the manufacturer's instructions. The quality and quantity of the extracted RNA were evaluated using a TapeStation system (Agilent Technologies, USA) for RNA Integrity Number equivalent (RINe) values and a Qubit Flex Fluorometer (Thermo Fisher Scientific, USA) for measuring the RNA concentrations. RNA samples with an RINe value of ≥ 7.0 and a minimum quantity of 200 ng were selected for library preparation. rRNA) was depleted for library construction using an rRNA Depletion Kit (MGI Tech, China). The RNA was subsequently fragmented and reverse‐transcribed using an RNA Directional Library Prep Kit (MGI Tech, China). Libraries were prepared using a high‐throughput (rapid) Sequencing Kit (MGI Tech, China). Transcriptome sequencing was conducted on the MGI DNBSEQ G400‐RS platform at the Kyungpook National University (KNU) NGS Core Facility (Daegu, South Korea). After sequencing, the reads were filtered using SOAPnuke to retain only those with a Phred quality score of ≥ 30. Over 96% of the paired‐end reads from all samples passed the quality filtering process. High‐quality reads were aligned to the Mus musculus reference genome (GRCm39) using HISAT2. Subsequently, transcript assembly was conducted using StringTie2 based on the alignment data.

### DNA Sequencing for the Bacterial Community

Total DNA was extracted from mouse feces using a QIAamp PowerFecal Pro DNA Kit (QIAGEN, Germany), following the manufacturer's instructions. A Qubit Flex Fluorometer (Thermo Fisher Scientific) was used to quantify the concentration of the extracted DNA. The 16S rRNA V4 region was amplified by using the forward primer attached to a 5′ Illumina adapter (5′‐barcoded‐GTGNCAGCMGCCGCGGTRA‐3′) and the indexed reverse primer (5′‐barcoded‐GACTACNVGGGTWTCTAATC‐3′). The quantity and quality of the amplicon libraries were measured using a Qubit Flex Fluorometer (Thermo Fisher Scientific) and Agilent D1000 ScreenTape System (Agilent Technologies), respectively. Each library was sequenced using the Illumina MiSeq platform with MiSeq Reagent Kit v2 (300‐cycle kits) at the KNU NGS Core Facility (Daegu, South Korea).

Raw reads were processed using Quantitative Insights into Microbial Ecology 2 (QIIME2; ver. 2024.5).^[^ ^41^ ^]^ Low‐quality sequences (<Q30) were excluded from analysis. DADA2 was utilized to trim and denoise the cleaned reads.^[^ ^42^ ^]^ Amplicon sequence variants (ASVs) were identified at the microbial taxonomic level using the SILVA database (ver. 138) and a naïve Bayesian pre‐trained QIIME2 classifier.^[^ ^41^ ^]^ The mitochondrial, chloroplast, and unassigned sequences were removed from the evaluated ASVs. The processed reads were subsequently repurified to a depth of 1500 reads.

### Quantification of Targeted Genes

Quantitative real‐time polymerase chain reaction (qRT‐PCR) was used to quantify target gene expression. Each reaction included 800 ng of template DNA, specific primers, and 2X QGreenBlue qPCR Master Mix (CellSafe, Seoul, Korea). Amplification was performed in a Bio‐Rad thermal cycler (Hercules, CA, USA) under the following conditions: initial denaturation at 95 °C for 3 min, followed by 40 cycles of 95 °C for 10 s (denaturation), 58 °C for 10 s (annealing), and 72 °C for 10 s (extension with fluorescence detection). A melting curve analysis was conducted at the end of the reaction to confirm the amplification specificity. Relative expression levels of target genes (Table S2, Supporting Information) were determined using the 2–∆∆Ct method, with GAPDH as the internal control.

### Statistical Analysis

Statistical analyses were performed using GraphPad Prism version 10.5.0. (GraphPad Software, San Diego, CA, USA). All data were presented as mean ± standard error of the mean (SEM). Unpaired *t*‐tests were conducted to evaluate differences between groups at each time point. Statistical significance was defined as p < 0.05 and was indicated using the commonly accepted asterisk notation: *p* < 0.05 (^*^), *p* < 0.01 (^**^), and *p* < 0.001 (^***^). All experiments were independently repeated at least three times.

Statistical analyses were conducted using R (v4.0.2;) for the microbiome data. The vegan (v2.6‐4) and phyloseq (v1.30.0) R packages were used for principal coordinate analysis (PCoA). The PCoA was performed using ASVs and the Bray‐Curtis dissimilarity distance matrix. Permutational multivariate analysis of the variance was used to compare the different gut microbiota groups.

## Conflict of Interest

The authors declare that they have no known competing financial interests or personal relationships that could have appeared to influence the work reported in this paper.

## Supporting information



Supporting Information

## Data Availability

The data that support the findings of this study are available from the corresponding author upon reasonable request.
