# Supplementary material for: Chronic PET‐Microplastic Exposure: Disruption of Gut–Liver Homeostasis and Risk of Hepatic Steatosis
Source: Adv Sci (Weinh). 2025 Oct 22;13(1):e12030. doi: 10.1002/advs.202512030 (PMC12766997; doi:10.1002/advs.202512030)
Supplement: Supplementary file 1 — Supporting Information [file ADVS-13-e12030-s001.docx]

**Supporting information**

**Table S1**. Particle size distribution of polyethylene terephthalate (PET). PET-MP were generated by milling commercially available PET water bottles manufactured in Korea. The milled material was dry-sieved through a 500-µm stainless-steel mesh to collect particles <500 µm. A 1.0 g aliquot of the fraction was analyzed by laser diffraction (Mastersizer 3000, Malvern Panalytical, UK) in dry dispersion mode at 3 bar gauge pressure. Measurements were performed in triplicate. Data are presented as % volume in each size range. Detailed descriptions have been included in the Methods section of the Supplementary Materials

| Size (μm) | % Volume In |
| --- | --- |
| < 1 | 1.11 |
| 1-10 | 6.14 |
| 10-100 | 59.96 |
| 100-200 | 25.84 |
| 200-400 | 6.93 |

**Table S2. Primer sequences for target genes**

| Name | Forward | Reverse |
| --- | --- | --- |
| Tnfα | GGTGCCTATGTCTCAGCCTCTT | GCCATAGAACTGATGAGAGGGAG |
| Il-6 | TACCACTTCACAAGTCGGAGGC | CTGCAAGTGCATCATCGTTGTTC |

**Table S3.** Quantification of polyethylene terephthalate (PET) in liver tissue of CON and MP groups. Approximately 1 g of liver tissue was digested with 5 mL of 10% KOH at 45 °C for 72 h in a shaking incubator. The suspension was sequentially filtered through 20 µm and 1 µm stainless-steel filters to isolate MPs in the 20–1 µm range. Retained materials on the filters were analyzed by pyrolysis–gas chromatography/mass spectrometry (Py–GC/MS) using a multi-shot pyrolyzer (Py-3030D, Frontier Lab) coupled to a GC/MS system (Agilent 8890A/5977C). PET was identified and quantified based on the extracted-ion chromatographic peak area of benzoic acid (m/z 105), a characteristic pyrolysate of PET.

(unit: ug)

| Polymer | LOD | Blank | Experimental groups | |
| --- | --- | --- | --- | --- |
|  |  |  | CON | MP |
| PET | 0.391 | n.d. | n.d. | n.d. |

n.d.: not detected


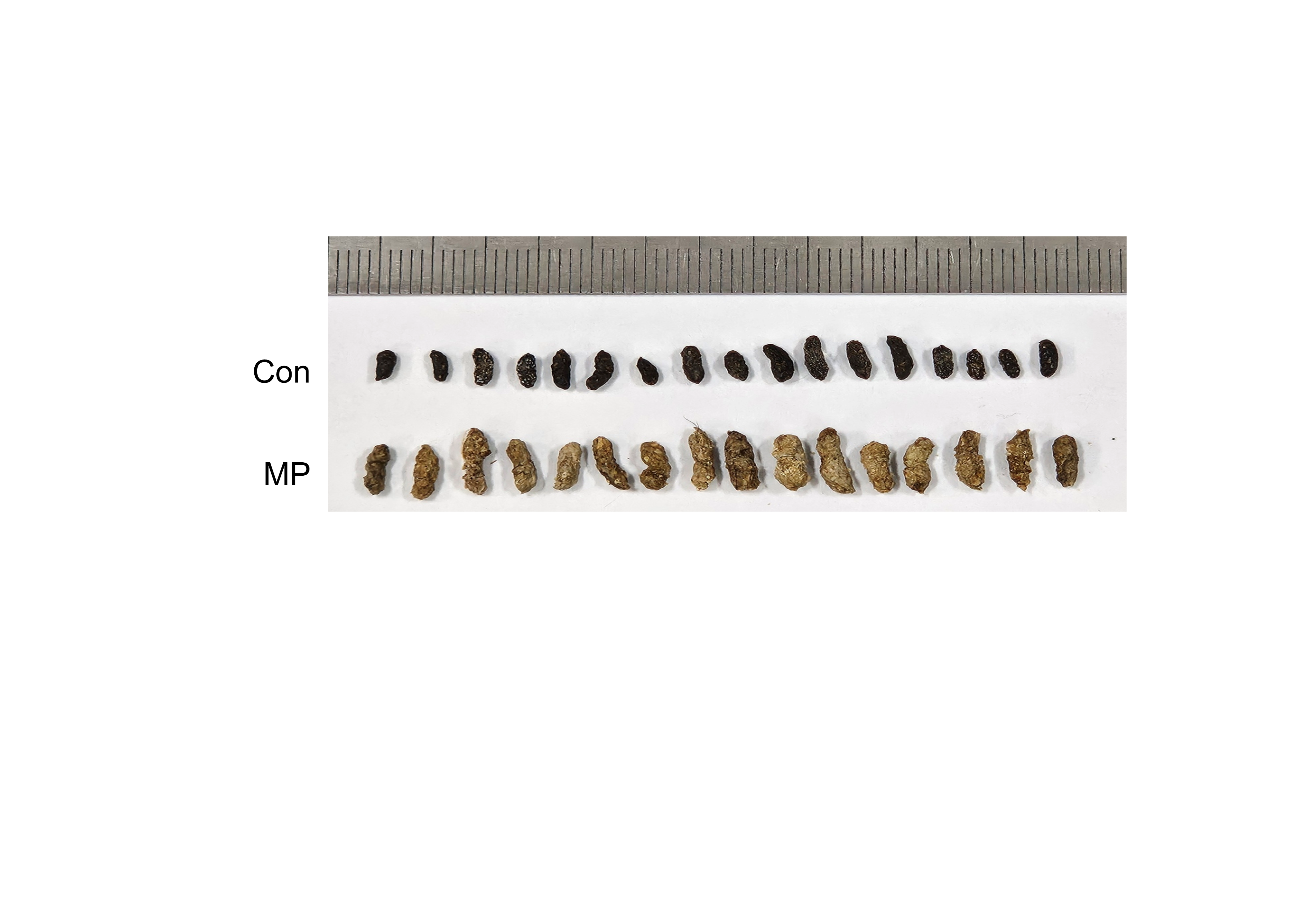


**Fig. S1**. Representative images of fecal pellets collected from Con group (top row) and MP group (bottom row) mice.
Visual comparison shows distinct differences in fecal morphology, including pellet size, shape, and color. Feces from Con are generally smaller and darker, whereas those from MP appear larger, lighter in color, and more irregular in shape. A scale ruler is shown at the top for size reference.


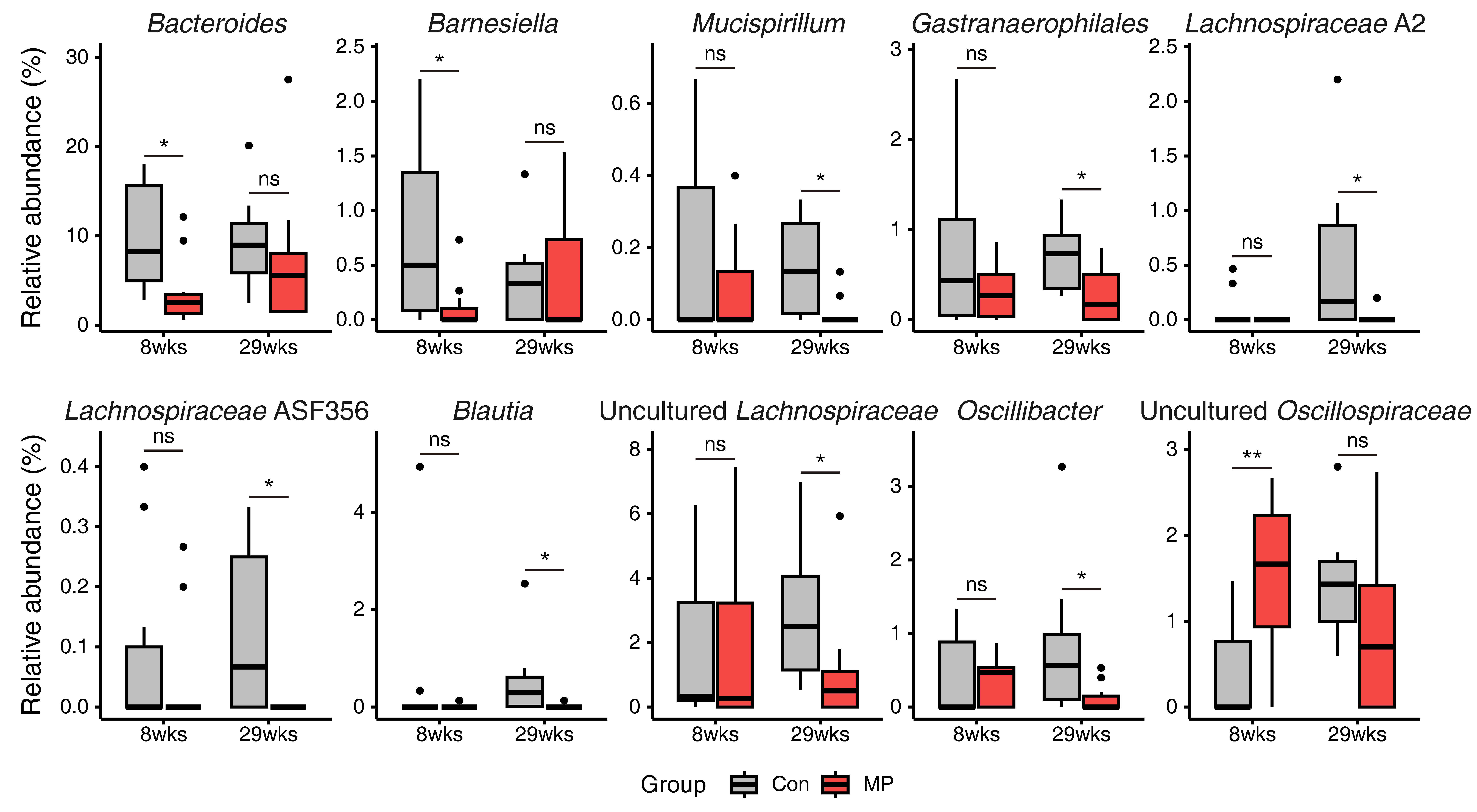


**Fig. S2.** Differential genera in mice following MP ingestion at 8 and 29 weeks.

Boxplots show differentially abundant genera between Con and MP groups at each time point. Unpaired t-tests were conducted to evaluate differences between groups at each time point. Statistical significance was defined as p < 0.05 and is indicated using the commonly accepted asterisk notation: *p < 0.05, **p < 0.01, and ***p < 0.001. All experiments were independently repeated at least three times.

| (A)  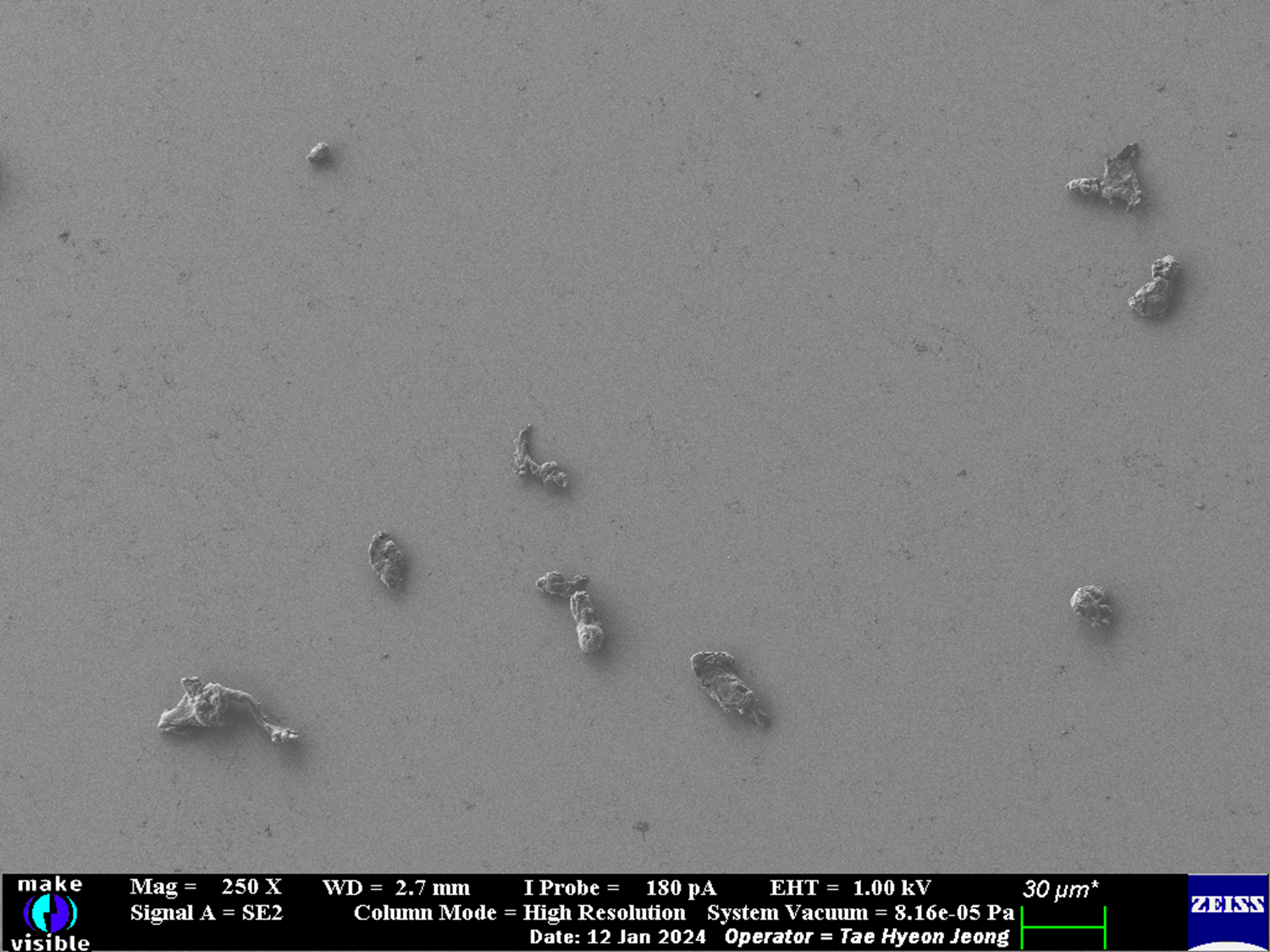 | (B)  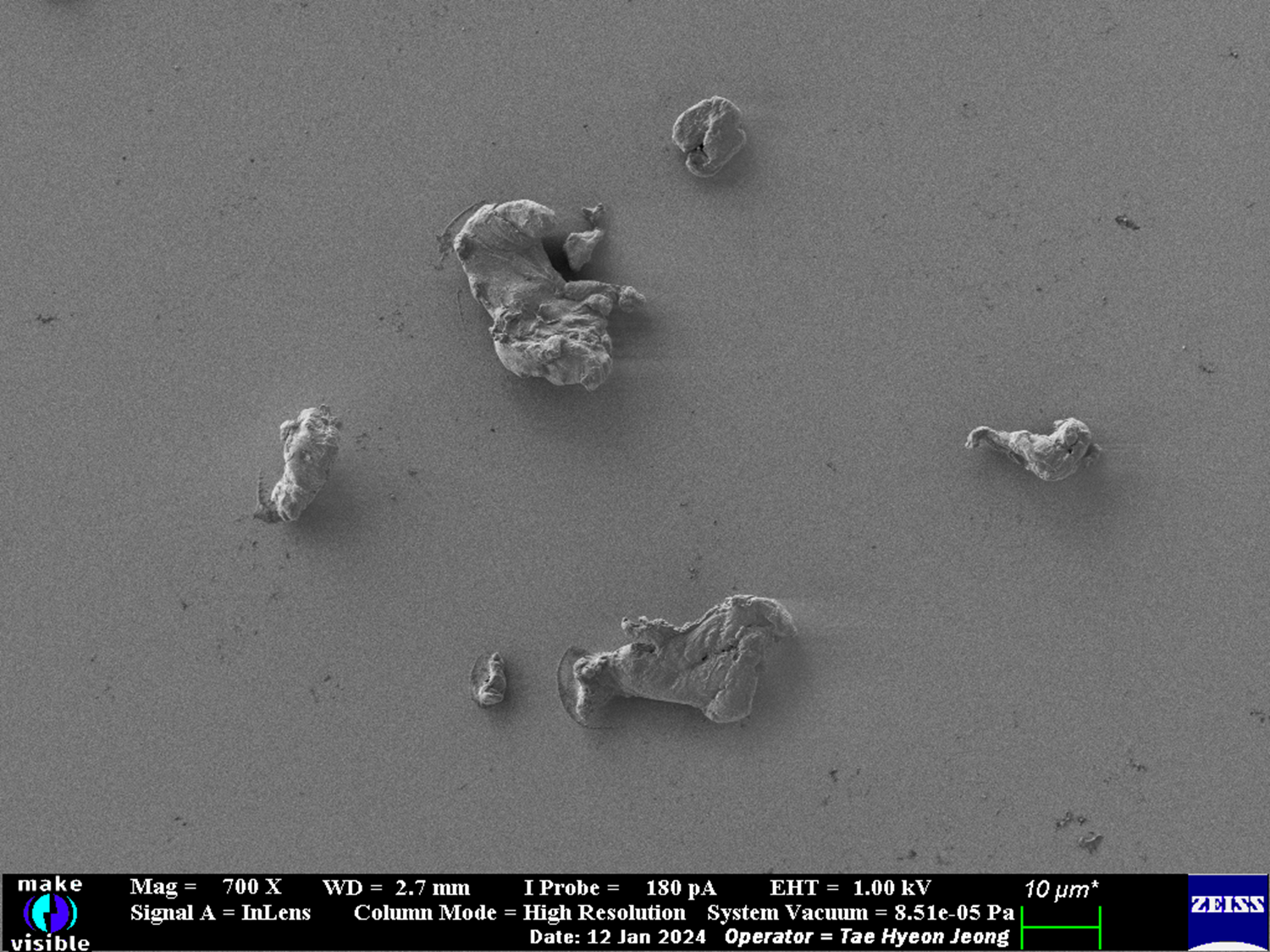 |
| --- | --- |

**Fig. S3.** Scanning electron microscopy (SEM) images of mechanically abraded polyethylene terephthalate (PET)-MPsdy. Scale bars: (A) 30 µm; (B) 10 µm.

PET-MPs were prepared by mechanical abrasion of commercially available polyethylene terephthalate water bottles, followed by sieving to obtain particles smaller than 500 µm. For SEM observation, a small amount of dried PET-MP powder was evenly spread on double-sided carbon adhesive tape mounted on aluminum stubs. Loose particles were gently removed with a compressed air stream to minimize charging artifacts. Serial surface-view imaging by focused ion beam–scanning electron microscopy (FIB–SEM) was performed on a dual-beam system (Crossbeam 550; Zeiss Microscopy GmbH, Oberkochen, Germany). PET-MP samples were positioned at a stage height of 5 mm to place the specimen at the electron/ion-beam coincident point, and the stage was tilted to 54°. SEM measurements were acquired in triplicate on independently prepared specimens.

**Method for quantification of PET-MPs**

*Preparation and filtration of samples*

Approximately 1.0 g of each liver sample was digested with 5 mL of 10% (w/v) KOH in a 50 mL Erlenmeyer flask for 72 h at 45 °C with shaking at 120 rpm. The digest was vacuum filtered through a stainless-steel (SUS) membrane filter (21 mm diameter; 1 µm nominal pore size). The filter retaining the PET-MPs was folded and transferred into a deactivated pyrolyzer cup for subsequent analysis.

*Pyrolysis–GC/MS*

Analyses were performed on a pyrolyzer–GC/MS system comprising an EGA/PY-3030D pyrolyzer (Frontier Lab) coupled to an Agilent 8890 GC/5977 MS (Kwon et al., 2025). Cups containing standards or sample PET-MPs were introduced into the furnace preheated to 600°C; the interface was maintained at 320°C. Separation was achieved on a UA-5 metal capillary column (30 m × 0.25 mm i.d., 0.25 µm film). Helium served as the carrier gas at 1.0 mL/min (constant-flow mode). The GC inlet was maintained at 320°C with a split ratio of 50:1. The oven program was 40°C (2 min hold), ramped at 20 °C/min to 320°C, with a final hold of 14 min, yielding a total runtime of 30 min. The MS operated in full-scan mode (m/z 29–550) at 5.3 scans/sec, with the electron multiplier set to 974 V.

*Identification and Quantification*

Polymer identification was performed by spectral matching against the NIST 08 and Frontier Lab F-Search libraries. Quantification was carried out using the extracted-ion chromatogram (EIC) peak area of the characteristic indicator pyrolysate for each target polymer, with external calibration curves generated from reference microplastic materials processed under identical sample-preparation and instrumental conditions.

**Reference**

J. Kwon, H. Kim, M. Z. Siddiqui, H. S. Kang, J. H. Choi, S. Kumagai, & Y. M. Kim, *Food Chem.* **2025**, 467, 142193.
